# Supplementary material for: Diabetic control and compliance using glycated haemoglobin (HbA1C) testing guidelines in public healthcare facilities of Gauteng province, South Africa
Source: PLoS One. 2023 Aug 16;18(8):e0278789. doi: 10.1371/journal.pone.0278789 (PMC10431606; doi:10.1371/journal.pone.0278789)
Supplement: Attachment — Submitted filename: Response to Reviewers - HbA1C audit.docx [file pone.0278789.s001.docx]

**A REBUTTAL LETTER**

Dear Dr N Tlotleng,

We thank you for giving us the opportunity to submit a revised version of the manuscript titled “**Diabetic control and compliance using HbA1C testing guidelines in public healthcare facilities of Gauteng province, South Africa**”*.*

We are grateful for the time and effort taken by you as well as the reviewers to give valuable feedback on the above titled manuscript.

Sincerely,

Dr N Kone

Below is point-form response to the editor’s and reviewer’s comments.

Point by point responses to Editor’s comments:

- **Editor point 1**- **Please ensure that your manuscript meets PLOS ONE's style requirements, including those for file naming**.

**Response:** Thank you for providing us with the relevant templates for the requirements of PLOS ONE for research report. We have changed all major headings to font size 18 and also wrote them into sentence case. Figure captions changed as per PLOS ONE requirements.

- **Editor point 2** - **Please provide additional details regarding participant consent. In the ethics statement in the Methods and online submission information, please ensure that you have specified what type you obtained (for instance, written or verbal, and if verbal, how it was documented and witnessed). If your study included minors, state whether you obtained consent from parents or guardians. If the need for consent was waived by the ethics committee, please include this information.**

**Response**: This study didn’t require patients’ consent as it used retrospective data from the Corporate Data Warehouse (CDW) of the NHLS. We used patients’ unique identifier codes to anonymously match them to the data required for the study. It is with that background that the we received a written communication (email) from the Ethics committee of the University of Witwatersrand (HREC), granted permission to conduct the study. Patients below 18 years (minors), were excluded from the study (see p.6).

- **Editor point 3 - Thank you for stating the following financial disclosure: “The funders had no role in study design, data collection and analysis, decision to publish, or preparation of the manuscript”
  Response**: We agree to the statement “The funders had no role in study design, data collection and analysis, decision to publish, or preparation of the manuscript”. We didn’t require any funding as we used online retrospective data at no cost.

a) No funding was required/received.
b) “The funders had no role in study design, data collection and analysis, decision to publish, or preparation of the manuscript”.
c) Not applicable- there were no funders/funding needed/received for this study.
d) The statement - “The authors received no specific funding for this work.” has been added in the revised cover letter. We appreciate that you will change this on the online submission form on our behalf.

- **Editor point 4** - **Your ethics statement should only appear in the Methods section of your manuscript.**

**Response:** We have moved the ethics statement in the Methods section as requested (p.6).

- **Editor point 5 - In your Data Availability statement, you have not specified where the minimal data set underlying the results described in your manuscript can be found Authors must share the “minimal data set” for their submission. PLOS defines the minimal data set to consist of the data required to replicate all study findings reported in the article, as well as related metadata and methods. Additionally, PLOS requires that authors comply with field-specific standards for preparation, recording, and deposition of data when applicable.**

**Response:** The authors do not have permission to share the HbA1C data. Data is available from the National Health Laboratory Service Corporate Data Warehouse, subject to an application to the Academic Affairs and Research Office. This application can be made using the online system (<https://aarms.nhls.ac.za/>).

Point-By-Point Reply to Reviewer’s Comments

- **Reviewer #1:**

**Page 5:** **Reference 3 is incorrectly referenced.**

**Response:** We have amended reference 3, now reference 2 as: “Africa DoSoS. Mortality and causes of death in South Africa: Findings from death notification. Government Printers Pretoria; 2017” (p. 24).

**Page 8: PHC in full when used for the first time.**

**Response:** we have included the full name for PHC as “Primary Health Care”, when used for the first time (p.8).

**Figure 1 and page 10**: **The exclusion (inclusion) criteria included an age of ≥ 18 years.**

**Response**: We have reviewed and amended the criteria as “the inclusion criteria included an age ≥18”, (p.7, 10 and Figure 1).

**Page 10:** **Write in full POC when used for the first time.**
**Response:** POC has been written in full as “Point Of Care”, when used for the first time in the revised manuscript (p.8).

**Tables: n (%) not n= (%)**

**Response:** Where applicable, amendment has been made to use n (%) (p.11, 15, 16).
 **Figure 2A: Title, Patients classification according to HbA1C testing guidelines compliance (Fig 2A) and Correlation between testing guideline with sex, age, and facility type (Fig 2B). The Y axis indicates the percentage of samples reported**.

**Response:** Thank you very much for pointing it out, we have amended Figure 2 title to “Patients classification to HbA1C guidelines compliance and distribution by sex, age and facility type.” and the Y axis as “percentage of patients’ compliance for each category”.

**Page 18: Table 5. The adjusted hazard ratios for Indian/Asian and Coloured are lower than for Black Africans. Can this be due to sub-optimal performance in Black African populations as reported in many studies in US and South Africa? This needs to be discussed.**

**The adjusted hazard ratios for Hospital is higher when compared to PHC but hospital patients performed better. This is contradictory. Verify and justify.**

**Response:** The extended Cox proportional hazards model was used to determine factors associated with time to HbA1c control only for those patients entered into care with a first-ever out-of-control HbA1c and had at least two follow-up measurements. We reported adjusted hazard ratios, 95% confidence intervals and p-values. Indeed, ethnic differences in HbA1c concentrations have been noted in non-diabetic individuals, we have included and referenced it in the discussion section of the revised manuscript as well as the fact that “HbA1c may underestimate glycaemia in HIV positive people and overestimate glycaemia in people with TB (p.20).

With regards to the aHR for hospitalized patients, we analyze factors associated with time (time-related parameters) to attain HbA1C control for patients who started with an out-of-control HbA1C, thus a higher aHR implies good performance. Those who visit hospital for care are more likely to have in-control HbA1C compared to PHC. Possible reasons for this are management by diabetic specialists and access to second line therapy (p.18).

Discussion
 **The first paragraph of the discussion should present the main and novel findings before comparing them with others.**
**Response:** We appreciate the suggestions and have incorporated them in the first paragraph of the discussion (p.18).

**The third sentence of the first paragraph is irrelevant in that paragraph. It should constitute a paragraph with comparisons with other studies.**
**Response:** Thank you for bringing it to our attention, we have removed that sentence and moved it to the “Strengths and Limitations” section (p. 21).

**Page 20, paragraph 2**: **HbA1C test has been reported to perform sub-optimally in African populations (References from US and South African studies).**
**Response:** We have included the suggestion, supported by references from US and South Africa (p. 21).

**Include a section on the Strengths and limitations of the study before conclusion. The last paragraph under conclusion is on limitations but no strengths of the study. This should show the differential performance of HbA1C by ethnicity.**

**Response**: We have included a section on the strengths, with the limitations of the study before the conclusion, where we have inserted that the study shows that black Africans being the highest number tested, are also likely to have high number poor diabetic control (p. 21-22).

- **Reviewer #2:**

INTRODUCTION: **• It would be nice if you could include the following in the introduction section- the number of diabetes cases, the number of diabetic-related deaths reported, the prevalence of diabetes among different race groups and the projected number of diabetic cases in South Africa (If any latest report).**

**Response:** Thank you – that information is now included in the introduction (p. 5).

**• Is there any study conducted in a developing country like SA where patients obtained sufficient screening as per their guidelines? If it is, please include.**

In a KZN study, glycated haemoglobin was measured in 29.2% of patients once and 13.2% of patients twice in the past year. Evaluation of selected records demonstrated compliance with the SEMDSA guidelines in only 4.2% of patients (p. 6).

**• Use either Type II diabetes or type 2 diabetes. “Type II diabetes mellitus” is used on page 5 line 4 and “type 2 diabetes” is used on page 3 line 5**

**Response:** We have amended on p. 3 and 5 to both read “Type 2 diabetes mellitus”.

RESEARCH DESIGN, MATERIALS AND METHODS:

**• The statistical analysis has been performed appropriately and rigorously**

**Response:** Thank you for your comment.

**• Statistical analysis: Please include the statistical software platform that you used (SAS and Stata SE) under the heading Statistical analysis**

**Response:** Done as requested, the software platform is now included under the statistical analysis heading (p.9).

**• Page 6 line 9: change Central data warehouse (CDW) to “Central Data Warehouse (CDW)”**

**Response:** we have changed Central data warehouse (CDW) to “Central Data Warehouse (CDW)”, when used for the first time (p.6).

**• Page 8 line 10: PHC full form is not given (used for the first time)**

**Response:** PHC has been written in full as “Primary Health Care” where it was used for the first time (p.8).

**• Use either HbA1C or HbA1c. For example, is page 7 line 2 -HBA1C and lines 7, 8 and 9-HbA1c.**

**Response:** HbA1C is the adopted format in the revised manuscript.

**• Page 10 line 3: Kaplan Meier to “Kaplan-Meier”**

**Response:** Amendment to “Kaplan- Meier” has been done (p.3, 10 and figure 3).

RESULTS

**• Figure 1: Exclusion criteria are given in brackets Under Met Inclusion Criteria. Please check.**

**Response:** We amended the legend to “inclusion” criteria, corresponding to the “inclusion” criteria in the figure (Figure 1 and p.7, 10).

**• HbA1c category: The HbA1c category given on page 9 line 9 is ≤7, >7 - ≤9 and >9% and on Page 10 line 16 <7%, >7-≤9% and >9%. Please check.**

**Response:** Both pages now read “<7%, >7-≤9% and >9%”.

**• Please check the grouping categories given in Table 3**

**Response:** We have added a heading to show “good controls” in the HbA1C category and Median follow-up period columns (Table 3, p.14).

**• Figure 2 is unclear**

**Response:** Figure 2 title and legend have been amended**.**

**• Page 16: last sentence. Is it a clinic or PHC? Also, in Figure 3C, please check.**

**Response:** The sentence has been changed to PHC. (“Primary Health Care” usually includes clinic(s)).

**• Page 19 Line 17 please check if this is a correct statement “It has been reported that reduced frequency of HbA1c testing is associated with poor control”**

**Response:** Yes, the statement is correct (p. 18-19; reference 18, 19).

CONCLUSION

**• The data support the conclusions.**

**Response:** Thank you for the positive feedback

**• 2nd paragraph can be separated as “Limitations of the study”**

**Response:** We have grouped the limitations with the strengths of the study and moved them at the end of the discussion section (p.21, 22).

**Response:** Thank you for the positive feedback and highlighting the questions above.
